# Supplementary material for: The Effects of SRT1720 Treatment on Endothelial Cells Derived from the Lung and Bone Marrow of Young and Aged, Male and Female Mice
Source: Int J Mol Sci. 2021 Oct 14;22(20):11097. doi: 10.3390/ijms222011097 (PMC8540697; doi:10.3390/ijms222011097)
Supplement: Supplementary file 1 [file ijms-22-11097-s001.zip › ijms-1410497-supplementary.pdf]

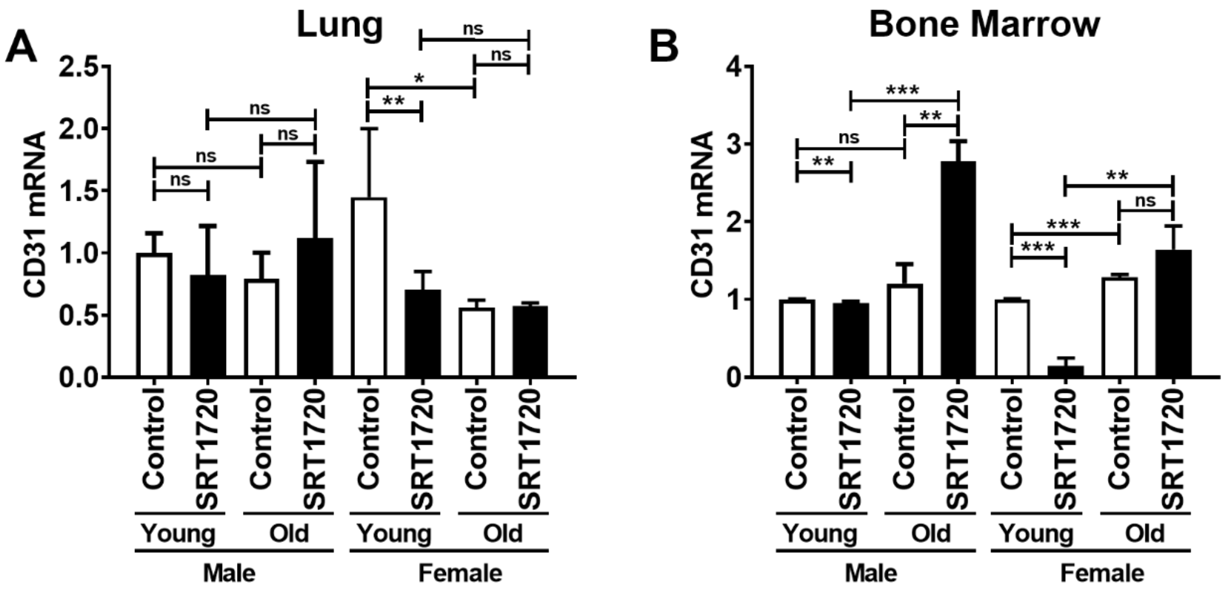

Supplementary Figure S1. Real-time PCR analysis of ECs isolated from lungs (**A**) or bone marrow (**B**). Relative mRNA expression was measured for CD31. Young male control (vehicle treated) expression was set to 1.0 and expression in all other samples are shown relative to the young male control. Data are expressed as the mean ± SD. An unpaired Student's t-test was used to identify statistically significant differences. ns = not significant, **p* < 0.05, ***p* < 0.01, ****p* < 0.001, and *****p* < 0.0001.

Bone Marrow

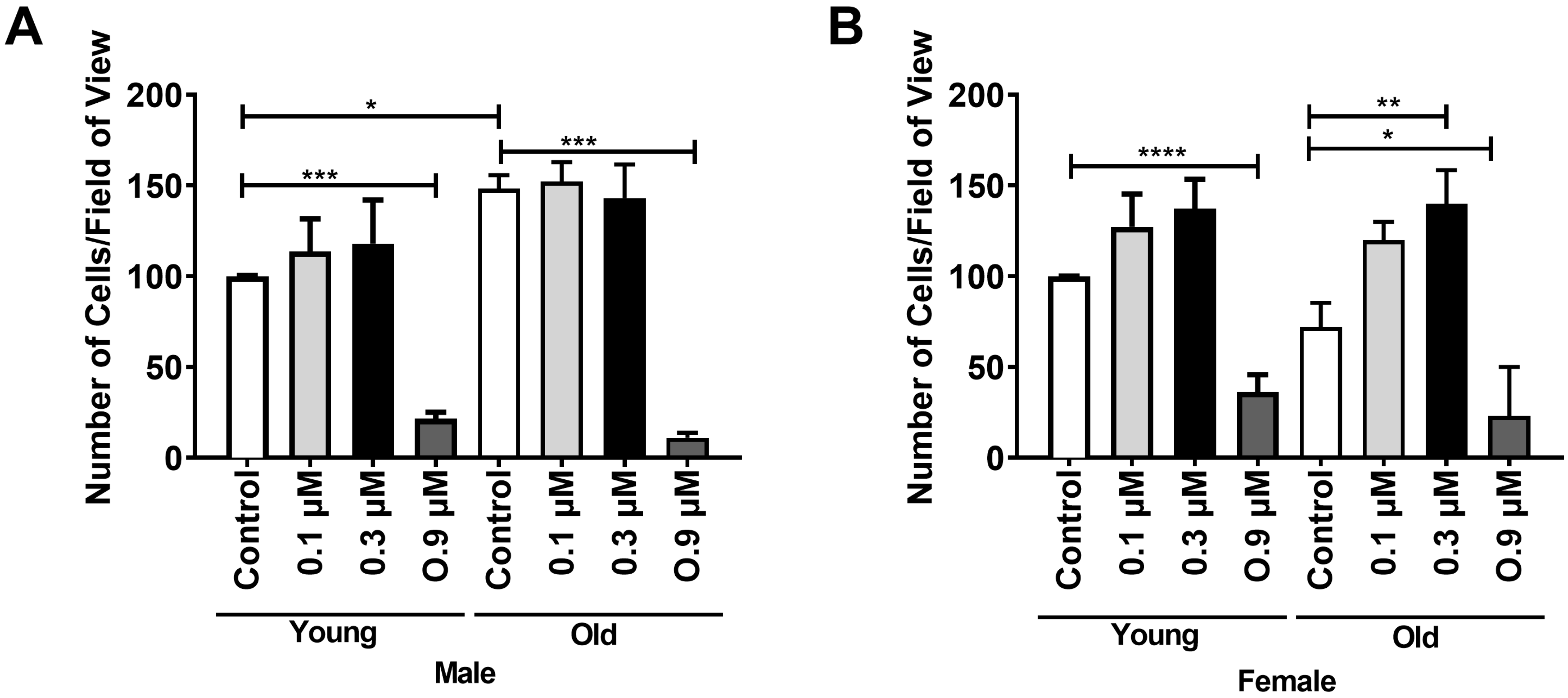

Supplementary Figure S2. Proliferation of bone marrow ECs isolated from both male (**A**) and female (**B**) treated with multiple concentrations of SRT1720 (0, 0.1, 0.3, and 0.9 μ M). Data are expressed as the mean ± SD. Young male and female controls (vehicle treated) parameters were set to 100.0 and parameters in all other samples are shown relative to the young male controls. An unpaired Student's t-test was used to identify statistically significant differences. ns = not significant, *p < 0.05, **p < 0.01, ***p < 0.001, and ****p < 0.0001.
